# Bioinformatics, Computational Informatics, and Modeling Approaches to the Design of mRNA COVID-19 Vaccine Candidates

**DOI:** 10.3390/computation10070117

**Published:** 2022-07-08

**Authors:** Olugbenga Oluseun Oluwagbemi, Elijah K. Oladipo, Olatunji M. Kolawole, Julius K. Oloke, Temitope I. Adelusi, Boluwatife A. Irewolede, Emmanuel O. Dairo, Ayodele E. Ayeni, Kehinde T. Kolapo, Olawumi E. Akindiya, Jerry A. Oluwasegun, Bamigboye F. Oluwadara, Segun Fatumo

**Affiliations:** 1 Department of Computer Science and Information Technology, Faculty of Natural and Applied Sciences, Sol Plaatje University, Kimberley 8301, South Africa; 2 Department of Mathematical Sciences, Stellenbosch University, Stellenbosch 7602, South Africa; 3 National Institute of Theoretical and Computational Sciences (NiThECs), Stellenbosch 7602, South Africa; 4 Laboratory of Molecular Biology, Immunology and Bioinformatics, Department of Microbiology, Adeleke University, Ede 232104, Nigeria; 5 Genomics Unit, Helix Biogen Institute, Ogbomoso 210214, Nigeria; 6 Department of Microbiology, University of Ilorin, Ilorin 234031, Nigeria; 7 Department of Natural Science, Precious Cornerstone University, Ibadan 200223, Nigeria; 8 Computational Biology/Drug Discovery Laboratory, Biochemistry Department, Ladoke Akintola University of Technology, (LAUTECH), Ogbomoso 210214, Nigeria; 9 Department of Virology, College of Medicine, University of Ibadan, Ibadan 200132, Nigeria; 10 Department of Medical Microbiology and Parasitology, University of Ibadan, Ibadan 200132, Nigeria; 11 Microbiology Programme, Department of Biological Science, Olusegun Agagu University of Science and Technology, Okitipupa 350113, Nigeria; 12 The African Computational Genomics (TACG) Research Group, MRC/UVRI and LSHTM, Entebbe 7545, Uganda; 13 Department of Non-Communicable Disease Epidemiology (NCDE), London School of Hygiene and Tropical Medicine, London WC1E 7HT, UK

**Keywords:** bioinformatics, COVID-19, SARS-CoV-2, immunoinformatic, mRNA, vaccine, modeling, computational

## Abstract

This article is devoted to applying bioinformatics and immunoinformatics approaches for the development of a multi-epitope mRNA vaccine against the spike glycoproteins of circulating SARS-CoV-2 variants in selected African countries. The study’s relevance is dictated by the fact that severe acute respiratory syndrome coronavirus 2 (SARS-CoV-2) began its global threat at the end of 2019 and since then has had a devastating impact on the whole world. Measures to reduce threats from the pandemic include social restrictions, restrictions on international travel, and vaccine development. In most cases, vaccine development depends on the spike glycoprotein, which serves as a medium for its entry into host cells. Although several variants of SARS-CoV-2 have emerged from mutations crossing continental boundaries, about 6000 delta variants have been reported along the coast of more than 20 countries in Africa, with South Africa accounting for the highest percentage. This also applies to the omicron variant of the SARS-CoV-2 virus in South Africa. The authors suggest that bioinformatics and immunoinformatics approaches be used to develop a multi-epitope mRNA vaccine against the spike glycoproteins of circulating SARS-CoV-2 variants in selected African countries. Various immunoinformatics tools have been used to predict T- and B-lymphocyte epitopes. The epitopes were further subjected to multiple evaluations to select epitopes that could elicit a sustained immunological response. The candidate vaccine consisted of seven epitopes, a highly immunogenic adjuvant, an MHC I-targeting domain (MITD), a signal peptide, and linkers. The molecular weight (MW) was predicted to be 223.1 kDa, well above the acceptable threshold of 110 kDa on an excellent vaccine candidate. In addition, the results showed that the candidate vaccine was antigenic, non-allergenic, non-toxic, thermostable, and hydrophilic. The vaccine candidate has good population coverage, with the highest range in East Africa (80.44%) followed by South Africa (77.23%). West Africa and North Africa have 76.65% and 76.13%, respectively, while Central Africa (75.64%) has minimal coverage. Among seven epitopes, no mutations were observed in 100 randomly selected SARS-CoV-2 spike glycoproteins in the study area. Evaluation of the secondary structure of the vaccine constructs revealed a stabilized structure showing 36.44% alpha-helices, 20.45% drawn filaments, and 33.38% random helices. Molecular docking of the TLR4 vaccine showed that the simulated vaccine has a high binding affinity for TLR-4, reflecting its ability to stimulate the innate and adaptive immune response.

## 1 Introduction

Severe acute respiratory syndrome (SARS) and Middle East respiratory syndrome (MERS), of the viral coronavirus family, have ravaged the world in the last two decades [1]. The World Health Organization (WHO, henceforth) declared COVID-19 a global pandemic. This declaration was made open in the year 2020. As of 20 January 2022, 336,790,193 cases of COVID-19 and 5,560,718 deaths [2] were confirmed. The RNA of the virus SARS-CoV-2, of the family coronaviridae, possesses a spike (S) glycoprotein, which extends over the surface of the virus to initiate the insemination of coronavirus into the host cells [3,4]. On this glycoprotein, there are 14 residue-binding receptors, which communicate with the angiotensin-converting-enzyme 2 (ACE2) receptor [5]. Coronavirus spike glycoprotein has acceptable antigenicity and immunogenicity [3,6].

As of 5th of August 2021, over 6000 deaths were recorded within a week, with 19% increases in the confirmed cases of SARS-CoV at almost 300,000 [7,8]. About 6000 delta variants have been recognized in more than 20 countries in Africa, with South Africa having the greatest percentage [9].

Vaccine administration holds a great promise to successfully combat the menace of the COVID-19 global pandemic [10–12]. The adoption of messenger RNA (mRNA) in vaccine development is characterized with great flexibility. Messenger RNA encodes and expresses all types of proteins, and by rule it enables the production of vaccines for combating diverse diseases and protein replacement remedy [13]. The scientific significance of mRNA vaccine development cannot be overemphasized. The production of the Moderna and Pfizer/BioNTech COVID-19 vaccines followed this large-scale vaccine production pattern [14].

Messenger RNA vaccines provide a novel method of building immunity against pathogens [15]. One of the distinct roles of mRNA vaccines in the fight against SARS-CoV-2 is the provision of the blueprint of genes for the spike protein of COVID-19 [16]. Unlike peptide-based vaccines, mRNA vaccines do not have restraints of the MHC haplotype; however, as an advantage, mRNA vaccines have a self-adjuvanting property, which is lacking in protein-based vaccines. The mRNA also binds to pattern-recognition receptors [13]. The fundamental principle in the operations of the mRNA vaccines is to provide transcription, which assists in encoding wanted antigens. This is closely followed by the synthesis of RNA. The sequence that encodes the immunogens is present, and the technique can effortlessly be implemented for mRNA production [17].

Messenger-RNA-based vaccines have been found to have better biosafety characteristics in comparison to DNA-based vaccines because the translation of antigens and immunogens occurs in the cytoplasm instead of the nucleus. Therefore, it is almost impossible for mRNA to fuse into the host genome as opposed to DNA-based vaccines [10]. In addition, mRNA is safe for use as a vector in comparison with DNA because it conveys a small line of sequence for translation (a transient molecule) and does not communicate with the genes present in the host [10]. The methods of administering mRNA vaccines vary. Moreover, the effectiveness of the vaccine is sometimes influenced by its route of administration [18]. Furthermore, mRNA vaccines are effective and safe [18]. The most common method of administering the mRNA vaccine is by injection [19]. Available mRNA vaccines such as Moderna and Pfizer, 1–2 days after administering, have related side effects such as [16], pain, redness, fatigue, fever, myalgias, and arthralgias.

Immunoinformatics is an aspect of bioinformatics that is involved with the computational analysis of biological and immunological data; it also involves the designing of vaccine candidates by predicting the best usable antigens, adjuvant, carriers, and epitopes for a vaccine. Immunoinformatics approaches have reduced the needed time and cost for vaccine development [1].

The aim of this study is to apply an integrated knowledge of bioinformatics, computational informatics, and modeling approaches towards the design of mRNA COVID-19 vaccine candidates. Specifically, this study is aimed at designing a multi-epitope mRNA vaccine based on the genome sequences of circulating SARS-CoV-2 variants in Africa. The human leucocyte antigen (HLA) allele’s supertypes were also analyzed to ensure a wide population coverage for the designed vaccine. This is the scientific novelty of this research paper.

## 2 Materials and Methods

### 2.1 Study Design

**Figure 1:**
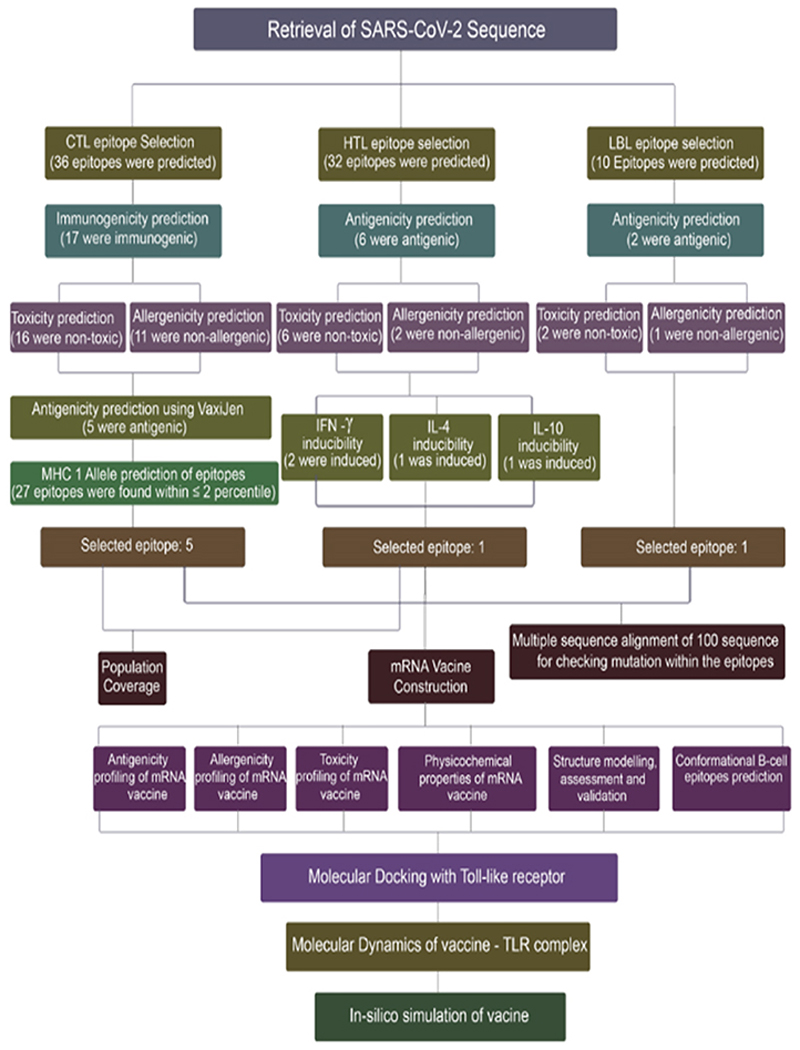
Systematic workflow diagram of the vaccine design for mRNA vaccine.

**Figure 2:**
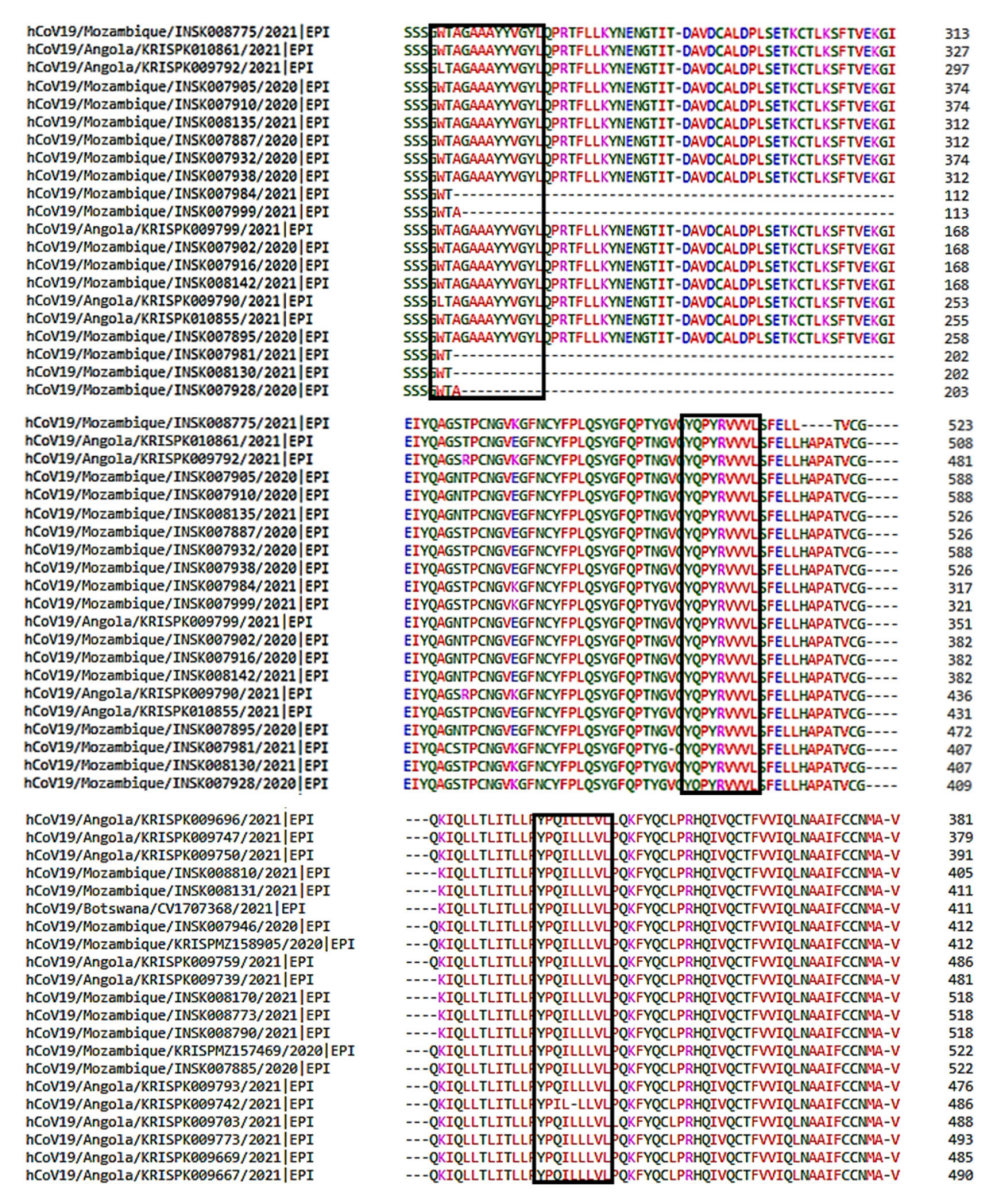
Multiple sequence alignment of spike glycoprotein sequences. The epitope sequences selected for vaccine design have been identified by boxes.

**Figure 3:**
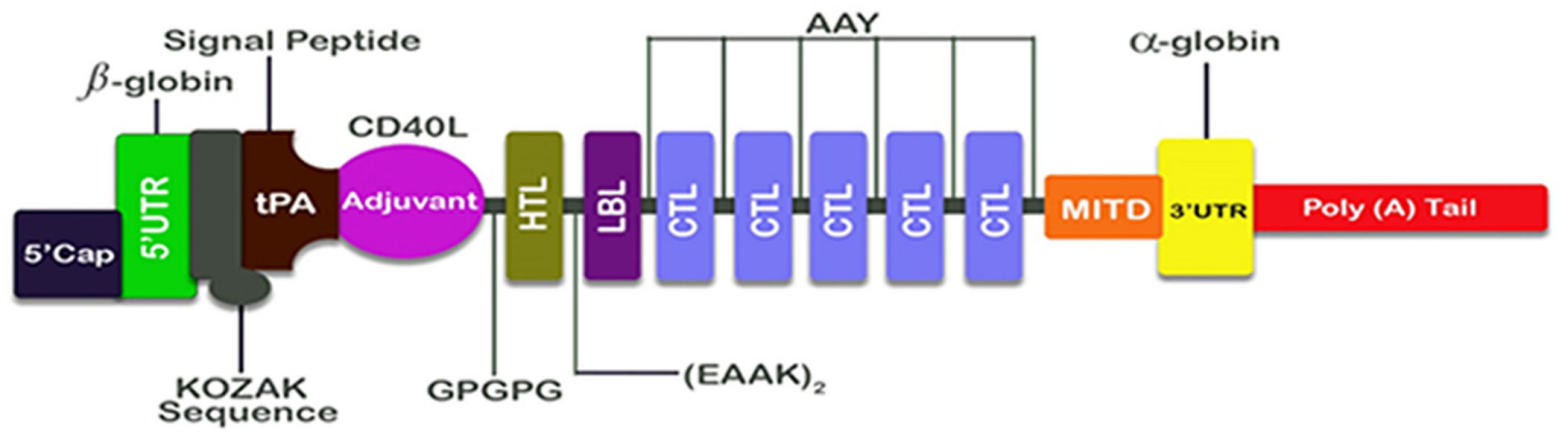
Scheme of the formulation of the mRNA vaccine against COVID-19.

The systematic workflow diagram for the mRNA vaccine design is shown in Figure 1. The design has 13 different sections, which are as follows: (1) retrieval of the whole genome sequences of SARS-CoV-2; (2) prediction and evaluation of CTL epitopes (See Table 1); (3) prediction and evaluation of HTL epitopes (See Table 1); (4) prediction and evaluation of LBL epitopes (See Table 1); (5) multiple sequence alignment (MSA) (See Figure 2); (6) docking between T-lymphocyte epitopes and MHC alleles (See Table 2); (7) population coverage prediction (See Table 3); (8) construction of mRNA vaccine (See Figure 3); (9) prediction of the toxicity, allergenicity, antigenicity, and physicochemical properties (See Table 4); (10) structure modeling, assessment, and validation; (11) conformational B-cell epitopes prediction; (12) molecular docking of vaccine with TLR receptor; (13) molecular dynamic simulation; (13) computational or *in silico* simulation.

### 2.2 Retrieval of SARS-CoV-2 Nucleotide Sequence

The data used for this research were retrieved from the Global Initiative for Sharing All Influenza Data (GISAID) database [20]. The data retrieved were targeted towards five African countries, namely Angola, Botswana, Mozambique, Lesotho, and Namibia. These data are the relevant genomic sequence data needed for the experiment and analysis. The data retrieved for these five African countries were based on criteria such as complete genome, low coverage exclusion, high coverage level, host, and date of submission. These criteria were considered for retrieving our sequences, and a total of 189 SARS-CoV-2 whole genome sequences deposited on the GISAID database between 1 December 2020 and 5 March 2021 were retrieved. Based on the criteria, three out of the five African countries in the study area—Angola (54), Botswana (26) and Mozambique (109)—had records for SARS-CoV-2 submitted to the GISAID database. There were no records found for the other two African countries (Lesotho and Namibia). The retrieved nucleotide sequences were annotated with the Wuhan reference sequence (accession number NC_045512.2) downloaded from NCBI database to establish the existing surface glycoprotein in the downloaded sequences.

### 2.3 Prediction and Evaluation of Cytotoxic T-Lymphocytes (CTL) Epitopes

Innate and adaptive defenses are some of the mechanisms utilized by host cells to neutralize viral replication [21]. One of such important adaptive defenses is the CD8+ cytotoxic T lymphocyte (CTL)’s response, which controls infection by a few mechanisms, along with the secretion of anti-viral cytokines and Fas/FasL-mediated lysis [21]. Many studies have featured CTL-mediated cytotoxicity, but the rate of fatality of virus-infected cells by CTL response in vivo is yet to be understood [21]. NetCTL v1.2 server was used to predict CTL epitopes [22]. A 9-mer CTL epitope was generated by the server for all the twelve major histocompatibility (MHC) class I supertypes available on its database, which includes A1, A2, A3, A24, A26, B7, B8, B27, B39, B44, B58, and B62, respectively, with the threshold set at 0.75. Epitopes predicted in the CTL supertypes for each country were inspected to determine overlapping epitopes. Epitopes having frequency overlap between 60 and above for each country were subjected for further analysis. Furthermore, the prediction of the CTL epitopes’ immunogenicity was performed by using the IEDB analysis resources. This tool showed negative and positive values. The epitope with the positive value was selected for further studies. Furthermore, Toxinpred [23] and AllerTOP 2.0 servers [24] were utilized to determine the toxicity and allergenicity of the immunogenic epitopes, respectively. Those that met the criteria were subjected to antigenicity assessment through the VaxiJen server [25]. Epitopes with value ≥ 0.5 were classified as antigenic. The antigenic epitopes were subsequently predicted for their MHC class I allelic partners by adopting the IEDB consensus algorithm, with human selected as the host.

### 2.4 Prediction and Evaluation of Helper T-Lymphocytes (HTL) Epitopes

The prediction of HTL epitopes is part of immunoinformatics approaches for vaccine development [1]. HTL aid the activity of other immune cells by binding to specific HTL epitopes using MHC class II molecules. Prospective HTL epitopes were predicted using IEDB MHC-II consensus algorithm [26]. Mouse was selected as the host species, and the epitopes were filtered using a percentile rank less than 0.25. The VaxiJen server was then used to compute the epitopes’ antigenicity [25]. Epitopes that were non-toxic and epitopes that were non-allergenic were selected by the ToxinPred and AllerTOP 2.0 servers [24]. After utilizing the IFNepitope [26], IL4pred [26], and IL10pred [27] servers, the remaining epitopes were assessed for interferon- (IFN-) inducibility and interleukin-4 (IL-4) and interleukin-10 (IL-10) inducibility. Three antigenic and cytokine-producing epitopes were shortlisted for the construction of the vaccine.

### 2.5 Projection and Evaluation of Linear B-Cells Lymphocytes (LBL) Epitopes

B cells form a core component of the adaptive immune system. One of their characteristics is the ability to identify and grant lasting protection against pathogens. They perform these functions by expressing proteins and producing antibodies on B cells’ surfaces. They identify antigens and bind to a section of an antigen in a very selective fashion. The knowledge of the identification procedure is adapted in vaccine design to develop more effective and long-lasting vaccines against pathogens [28]. IBCE-EL server [29] was utilized to predict the LBL epitopes in this study. Only epitopes that were positive were selected for the prediction by the server for probable LBL and occurrence of 25 times or more across the three countries. An in-house-developed R-script program was used to perform further analysis on the selected epitopes. The antigenicity of possible LBL epitopes was tested by applying the VaxiJen server [25]. The evaluation of the toxicity and the antigenic epitopes’ allergenicity was performed by using ToxinPred server and the AllerTOP 2.0 server.

### 2.6 Multiple Sequence Alignment of SARS-CoV-2 Nucleotide Sequence

Unlike DNA viruses, SARS-CoV-2 (an RNA virus) has great propensity of undergoing repetitive mutation [30]. To authenticate the probability of the selected region of our epitopes not having undergone mutation, 100 randomly selected spike glycoprotein sequences from the study area were fed into Clustal Omega software for analysis [31].

### 2.7 Designing of mRNA Vaccine

The methods adopted in the research conducted by Ahammad and colleagues [32] were applied to construct an mRNA against SARS-CoV-2 virus. The epitopes used for the vaccine construct were selected based on criteria such as the antigenicity, non-toxicity, non-allergenicity, and cytokine-inducing properties (HTL only). Linkers were used to link CTL, HTL, and LBL epitopes. These epitopes were selected to construct the mRNA vaccine. HTL and LBL epitopes were linked by using (EAAK)2. (EAAK)2 was used to space intra-LBL epitopes. AAY linker was used to link LBL and CTL epitopes. AAY linkers were also used to combine intra-CTL epitopes. Adjuvants play important roles in the design of effective vaccines for increased immunogenicity [33]. In this study, CD40 ligand (CD40L), a co-stimulatory molecule, which functions as an agonist to the human immune receptor by interacting with the antigen presenting cells [33], was utilized. CD40L sequence (UniProt ID: P29965) was retrieved from the UniProt database and putatively linked together with the HTL epitopes using the GPGPG linkers.

Furthermore, MHC I-targeting domain (MITD) was used to direct CTL epitopes to MHC I compartment of the endoplasmic reticulum and tissue plasminogen activator (tPA) [32]. The sequences of tPA (UniProt ID: P00750) and MITD (UniProt ID: Q8WV92) were retrieved from the Uniprot database. It is evident that instability has been a major problem encountered in the production of many mRNA-based therapeutics. Therefore, it is pertinent to include elements that naturally find expressions in eukaryotic mRNA, which is very important for mRNA stabilization [32]. We integrated the sequences of 5′ cap and poly (A) tail of our vaccine design with the sequence of 5′ and 3′ untranslated regions (UTRs) flanking its protein encoding ORF for mRNA molecules’ stability, accessibility of ribosomes, and interactions with the translation machinery [34]. It has been established that the length of poly (A) tail is significant in the translation efficiency of mRNA [32]. The length of the poly (A) tail utilized in our study was from 120–150 base long. This range has been considered as the optimal length according to existing studies. For instance, according to Ahammad and colleagues [32], Poly (A) tail functions effectively alongside 5′ m7G cap sequences within this range.

### 2.8 Prediction of Class I and Class II Epitopes’ Population Coverage

One of the important features of an epitope is its ability to closely bind to an HLA molecule. Human population coverage is another significant feature that is very crucial in selecting epitopes for vaccine design [35]. Population coverage is the expected percentage of individuals in a population having the likelihood of inducing an immune response to not less than one T-cell epitope in a set [36].

We utilized the IEDB population coverage tool [37] to determine the population of the screened epitopes and their MHC alleles. This tool was used to compute the distribution of persons predicted to respond to epitopes that have been selected with known HLA background [37]. The genotypic frequencies of HLA were also computed. The T-cell epitopes were queried based on certain variables such as area, ethnicity, and country [37]. The total global population was selected, and this was followed by the selection of the population associated with the subcontinents.

### 2.9 Vaccine Construct’s Predicted Antigenicity, Toxicity, Physicochemical Properties, and Allergenicity

One of the significant features considered essential in vaccine development is the propensity of designated vaccine candidates to possess antigenic property [33] and the capability of inducing immune response leading to the formation of B and T lymphocytes after administration. VaxiJen 2.0 and ANTIGENpro servers [38] were used for predicting the antigenicity of the final vaccine construct [25].

ANTIGENpro server is dependent on sequence, alignment free, and independent of pathogens in its predictive operations of protein antigenicity [38]. AllerTOP 2.0 [24] and AllergenFP 1.0 servers [39] were used to check the allergenicity of the final vaccine construct to determine if a vaccine construct is allergen or non-allergen.

AllerTOP’s prediction was based on ACC transformation and E-descriptors [24], while AllergenFP’s prediction was based on the classification of allergens and non-allergens datasets into five E-descriptors and then using auto-cross covariance (ACC) to transform its strings into uniform vectors [39]. ToxinPred server [23] was used to predict the vaccine construct’s toxicity. The operation of this server is dependent on the support vector machine (SVM) model. This helps in toxicity and non-toxicity classification [23]. An online web server, ExPASy ProtParam [40], was used to examine the physicochemical properties of the vaccine construct.

### 2.10 Prediction of the Secondary Structure of the Vaccine Construct

Protein secondary structure helps to determine the protein folding orientation [41]. SOPMA online server [42] was applied to assess the vaccine construct’s secondary structure [42]. The vaccine construct’s secondary structure prediction by SOPMA yielded an accuracy of 69.5%. Three-state structure (B-sheet, coil, and a-helix,) description was produced [43].

### 2.11 3D Structural Modeling, Assessment, and Validation

The vaccine construct’s 3D structure was evaluated using the Phyre2 server [44]. Phyre2 built 3D models by utilizing and applying remote homology detection techniques that have advanced capabilities. Phyre2 also assists with the prediction of binding sites for ligands and the analysis of amino acids variants’ effects [44].

Despite making use of advanced template-based methods for modeling the 3D structure of an unknown protein, inaccuracies may still exist within the model structure [45]. This is expected especially when the template proteins do not share enough corresponding homology with target proteins and thus may cause a deviation from the overall target structure due to differences in their sequences [45].

GalaxyRefine web server [46] was used to refine the vaccine construct’s 3D structure built by the Phyre2 server. The operations of the GalaxyRefine web server are as follows: rebuilding of side chains, repacking side chains, and the relaxation of structure by the molecular dynamics’ simulation. It has been proven that the web server approach has produced the best performance according to the assessment of CASP10 [46].

ProSA-web server [47] was used to validate the refined 3D model of the vaccine construct. This process helps to check the constructed 3D models of the vaccine structure for any potential errors. Some of the other applications of this web server include identification of errors in experimentally determined structures, protein engineering, and computation of the total score for specific input structure [47]. A structure is said to contain errors if its scores fall outside the score range of a native protein.

### 2.12 Prediction of Conformational B-Cell Epitope

ElliPro server was used for predicting [48] the conformational B-cell epitopes of the final vaccine 3D model structure. As a web-based tool, ElliPro can assist with predicting epitope antibodies inherent in a sequence’s protein antigens. It performs implementation of existing methods for protein structure as an ellipsoid. It computes protein residues’ protrusion indexes that lie outside ellipsoid [48].

### 2.13 Molecular Docking of Vaccine with TLR Receptor

Molecular docking is a significant bioinformatics technique widely adopted for the prediction of the binding affinity and orientation between a receptor and its ligand [32]. Our study examined the possible binding affinity between the vaccine’s construct and its receptor. ClusPro 2.0 server was used to conduct molecular docking [49]. There was molecular docking between the refined 3D model of the final vaccine construct and an immune toll-like receptor (TLR 4, PDB ID: 4G8A) obtained from Protein Data Bank [50].

### 2.14 Molecular Dynamics Simulations

The molecular dynamics was simulated by utilizing the iMODs server [51] to assess the physical movements and stability of the vaccine’s TLR4 docked complex. The iMOD server performs evaluation of protein stability by applying the normal mode analysis (NMA) towards the computation of the interior coordinates. The eigenvalue, elastic network model, covariance matrix, main-chain deformability plot, and B-factor values were used to depict the stability of the protein [51].

### 2.15 Immune Response Simulation

An assessment of the immunogenicity of all the predicted conjugate vaccine peptides and the attributes of the immune response was conducted by utilizing the C-ImmSim online server [52]. Associated immune interactions and epitopes are predicted by the server after utilizing a machine-learning-based technique. Three anatomical compartments are automatically simulated. These include: (i) bone, in a situation where there is stimulation of the hematopoietic stem cells, accompanied by the production of the myeloid cells, (ii) thymus, and (iii) lymphatic organ. The administering of three injections having the designed peptide vaccine was simulated at intervals of four weeks, i.e., day 0, day 28, and day 56. This prime–booster–booster method was adopted at an interval of 4 weeks to accomplish a lasting protective immune response.

Default parameter settings indicate the positioning at 1, 84, and 168. This implies that each time step has an interval of 8 h. Time step 1 depicts the administration of injection at time zero. There were administrations of three injections at intervals of four weeks. However, there were administrations of eight injections at four-week intervals to cause stimulation of repetitive reactions to the antigen. This scenario subjects the T-cell memory to continuous examination. The plot analysis provided a platform to graphically interpret the Simpson index [53].

## 3 Results

### 3.1 Prediction and Evaluation of CTL Epitopes

To obtain the best and choicest epitopes suitable for our vaccine construction from among the large number of CTL epitopes predicted across the three countries, an overlapping procedure was employed to avoid repetition of predicted epitopes.

There were 36 unique CTL epitopes of 12 MHC class I supertypes, with frequencies above 60 times predicted by the NetCTL v1.2 server [22]. Seventeen of the epitopes predicted, when subjected to evaluation, were positive for immunogenicity. IEDB class I immunogenicity tool was used for this evaluation. ToxinPred [23] and AllerTOP 2.0 servers [24] predicted that of the 17 immunogenic epitopes, 16 were non-toxic and 11 were non-allergenic. The 11 epitopes passed the three stages of prediction (i.e., immunogenicity, toxicity and allergenicity), and they were further assessed for antigenicity by utilizing the VaxiJen server [25]. The results predicted revealed that 5 out of the 11 epitopes were successful in scaling above the antigenicity threshold of 0.5, which were then selected for the vaccine construction (Table 1).

### 3.2 Prediction and Evaluation of HTL Epitopes

Predicted results from the IEDB MHC-II allele tool revealed that 32 distinctive epitopes with frequency of occurrence above 25 were predicted, and these spanned the three countries. Six of these epitopes assumed the VaxiJen threshold (≥0.5) for antigenicity. Results from the ToxinPred server revealed that the six antigenic epitopes were non-toxic [23]. AllerTOP 2.0 server predicted two epitopes to be non-allergenic [24].

Following a careful examination of the interferon-*γ* (IFN-*γ*), interleukin-4 (IL-4), and interleukin-10 (IL-10) inducibility using the IFNepitopes, IL4pred, and ILpred servers, respectively, there was only one epitope that fulfilled all the criteria (see Table 1).

### 3.3 Assessment and Prediction of LBL Epitopes

A rigorous assessment of the translated nucleotide sequences for the possible existence of B-cells epitopes was conducted using the BCpred server [54]. Epitopes that had percentile ranks higher than 0.9 were selected. Further evaluation was conducted for the possible existence of linear B cells by utilizing the IBCE-EL server. A total of 10 probable LBL epitopes were predicted. Two of the ten epitopes predicted for possible presence of LBL epitopes were proven to be antigenic (they met the criteria of being antigenic (≥0.5)) on the VaxiJen server. The two epitopes were predicted to be non-toxic by ToxinPred while one epitope was predicted to be non-allergic by the AllerTOP 2.0 server (see Table 1).

### 3.4 Multiple Sequence Alignment of SARS-CoV-2 Sequences

Multiple sequence alignment (MSA) of 100 spike glycoproteins of coronavirus was conducted by using Clustal Omega. Interestingly, no mutation was observed within the seven epitopes selected amongst the 100 SARS-CoV-2 spike glycoproteins (see Figure 2).

### 3.5 Designing of mRNA Vaccine

Analysis was performed on all seven selected epitopes (WTAGAAAYY, HRHLRFLTL, YQPYRVVVL, YPQILLLVL, SPRRARSVA, ISFHVLTKLRLKCKL, and WVFITTKTTKVG-WKVSSEF) to examine their interactions and their subsequent potentials for possible development of an mRNA vaccine construct. The 5′ Cap, 5′ UTR, Kozak sequence, and tPA (signal peptide) were merged with the adjuvant (CD40 ligand) and then linked to the HTL epitope by the assistance of the GPGPG linkers from the beginning of the N-terminal of the mRNA vaccine.

Epitopes were bonded together depending on their degree of interactional compatibility in a sequential manner with EAAKEAAK (HTL to LBL) and AAY (LBL to CTL and intra CTL) linkers, respectively. The AAY linkers were used to connect the C-terminal end to the CTL epitopes (see Figure 3).

### 3.6 Population Coverage

It is very important during epitope-based vaccine design to select epitopes that have diverse HLA binding specificities and to ensure a broad population coverage. This is particularly paramount because an epitope can effectively evoke an immunological response in individuals for cases that only find expression for a particular MHC molecule that forms a complex with it [35].

**Figure 4:**
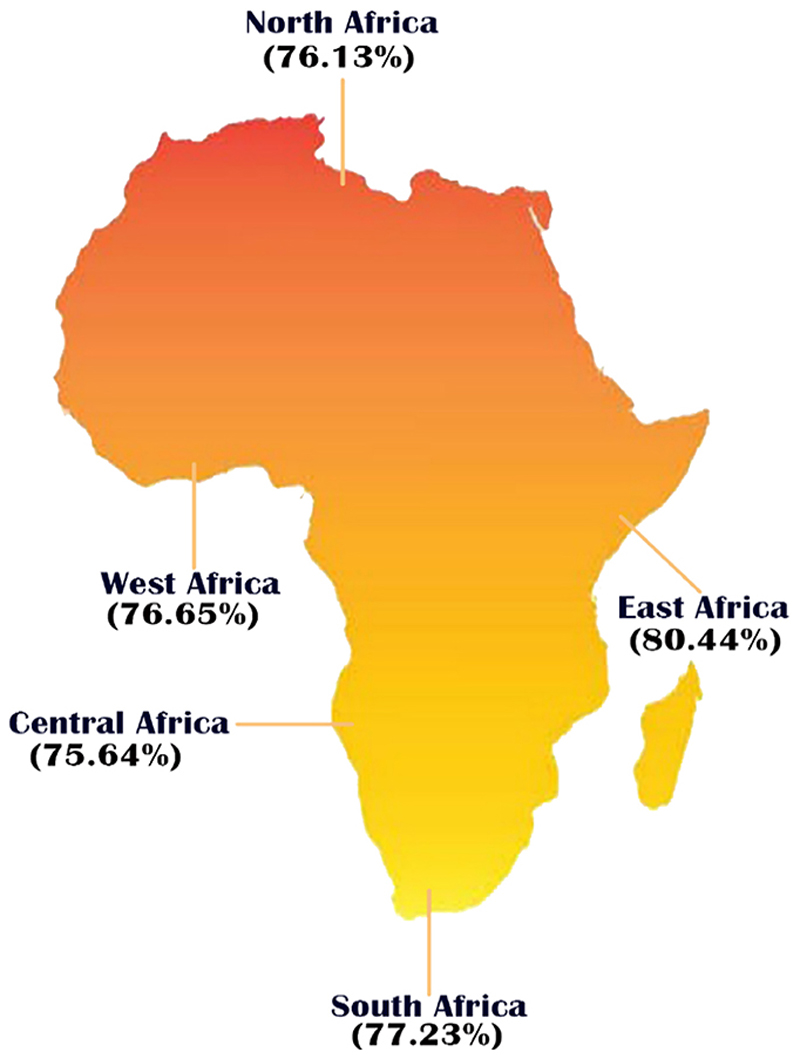
Population coverage of utilized T-lymphocyte epitopes. The highest region and the lowest region of coverage are, respectively, East Africa (80.44%) and Central Africa (75.64%).

Analysis on population coverage was performed with MHC class I and MHC class II epitopes and with associated HLA alleles within five African geographic regions found in the IEDB database. The result obtained revealed that all epitopes combined class I and II to have an average coverage of 77.22% (Table 3). Maximum coverage (80.44%) was found in East Africa, followed by South Africa (77.23%). West Africa and North Africa have a coverage of 76.65% and 76.13%, respectively, while Central Africa has the minimum coverage (75.64%), (See Table 3 and Figure 4).

### 3.7 Prediction of Allergenicity, Antigenicity, Physicochemical Properties, and Toxicity of the Vaccine Construct

The results of the allergenicity, antigenicity, physicochemical properties, and toxicity analyses are depicted in Table 4. The mRNA vaccine candidate’s antigenicity was examined using the VaxiJen and AntigenPro servers. Both servers revealed that the vaccine construct has high antigenicity scores of 0.5059 and 0.7334, respectively. These results indicate the vaccine’s ability to induce a robust immune response. Afterward, the vaccine’s allergenicity was evaluated by the Allertop server and the AllergenFP 1.0 server to determine its nature. The results revealed that the vaccine construct was non-allergenic and devoid of toxicity. Subsequently, the ExPaSy Protparam server [40] was applied to determine the vaccine’s physicochemical behavior. The results from the assessment of the vaccine construct’s physicochemical properties revealed that it consists of 1995 amino acids having a molecular weight of 223.1 kDalton. Results showed that the vaccine construct has a theoretical isoelectric point (pI) of 8.69. This indicates that it is slightly basic in nature.

The PH is defined by the theoretical pI in cases where the total charge of the peptide is zero and computed based on the pK of all amino acids’ resident in the peptides [54]. In addition, an index of 48.78 depicted an unstable vaccine construct. An instability score of 40 or less is a steady and a stable score [40]. Furthermore, the vaccine was characterized with a globular structure with 82.13 as its aliphatic index score. The vaccine construct has an estimated existence of 30 h in vitro for analysis in mammalian reticulocytes, more than >20 h in vivo in yeast, and >10 h in vivo for Escherichia coli, which connotes the stability of the vaccine’s in vitro and vivo phases. Furthermore, the coefficient of extinction was computed and was 273,135 M^−1^cm^−1^, with absorption values (0.1%) (g/1) 1.224 consisting of all cysteine pairs under aqueous conditions at 280 nm. The computed grand average of hydropathicity (GRAVY) score was −0.296. This shows that the vaccine construct is hydrophilic. It can foster interaction with water and blood and easily identify targets. It is clear from physicochemical analysis results that the vaccine construct’s contents meet the necessary criteria for vaccine formulation (See Table 4).

### 3.8 Secondary Structure Prediction

**Figure 5:**
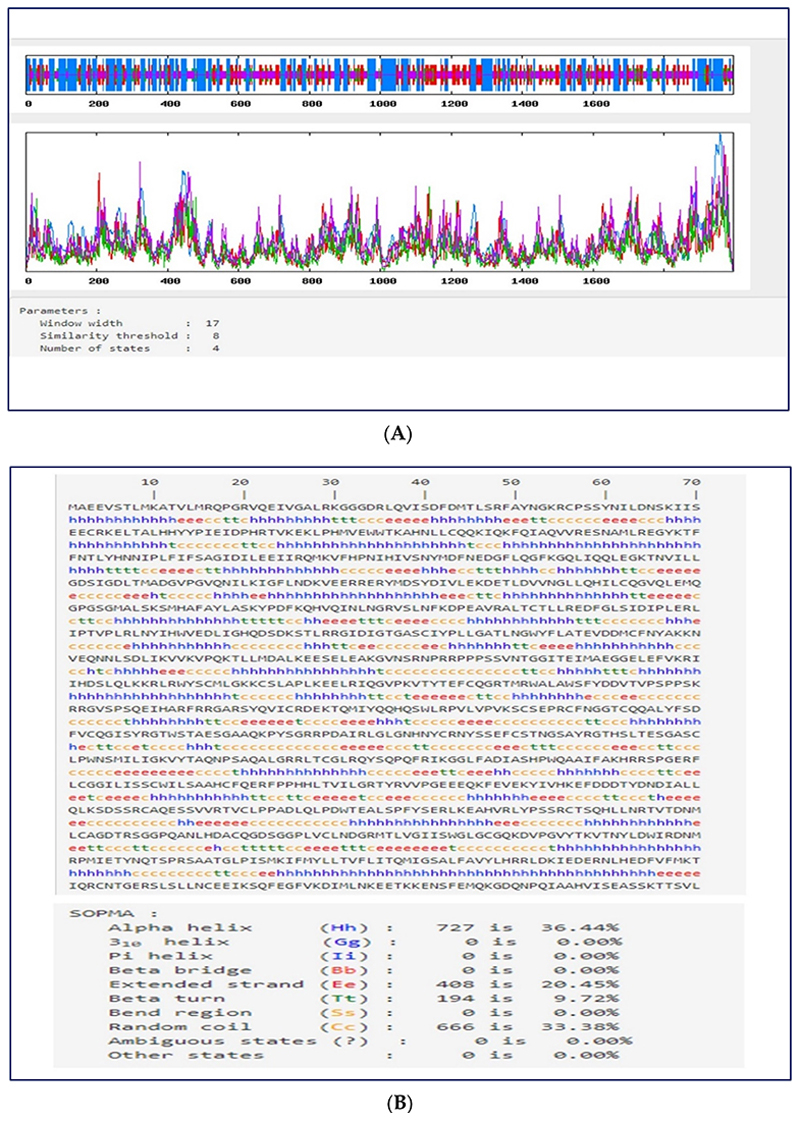
(**A**) Vaccine construct’s secondary structure. (**B**) Designed vaccine’s secondary structural analysis, revealing the fluctuations of its structural atoms, within a minimal range, depicting the stability of its structure.

The vaccine’s secondary structure was analyzed using the SOPMA online server [42]. The results obtained revealed a stabilized structure for the vaccine construct with 36.44% alpha-helix, 20.45% extended strands, and 33.38% random coils. This result also showed that the vaccine construct’s secondary structure is of good flexibility, stability, and globular conformation (see Figure 5A,B).

### 3.9 Three-Dimensional Structural Modeling, Refining, and Validation

**Figure 6:**
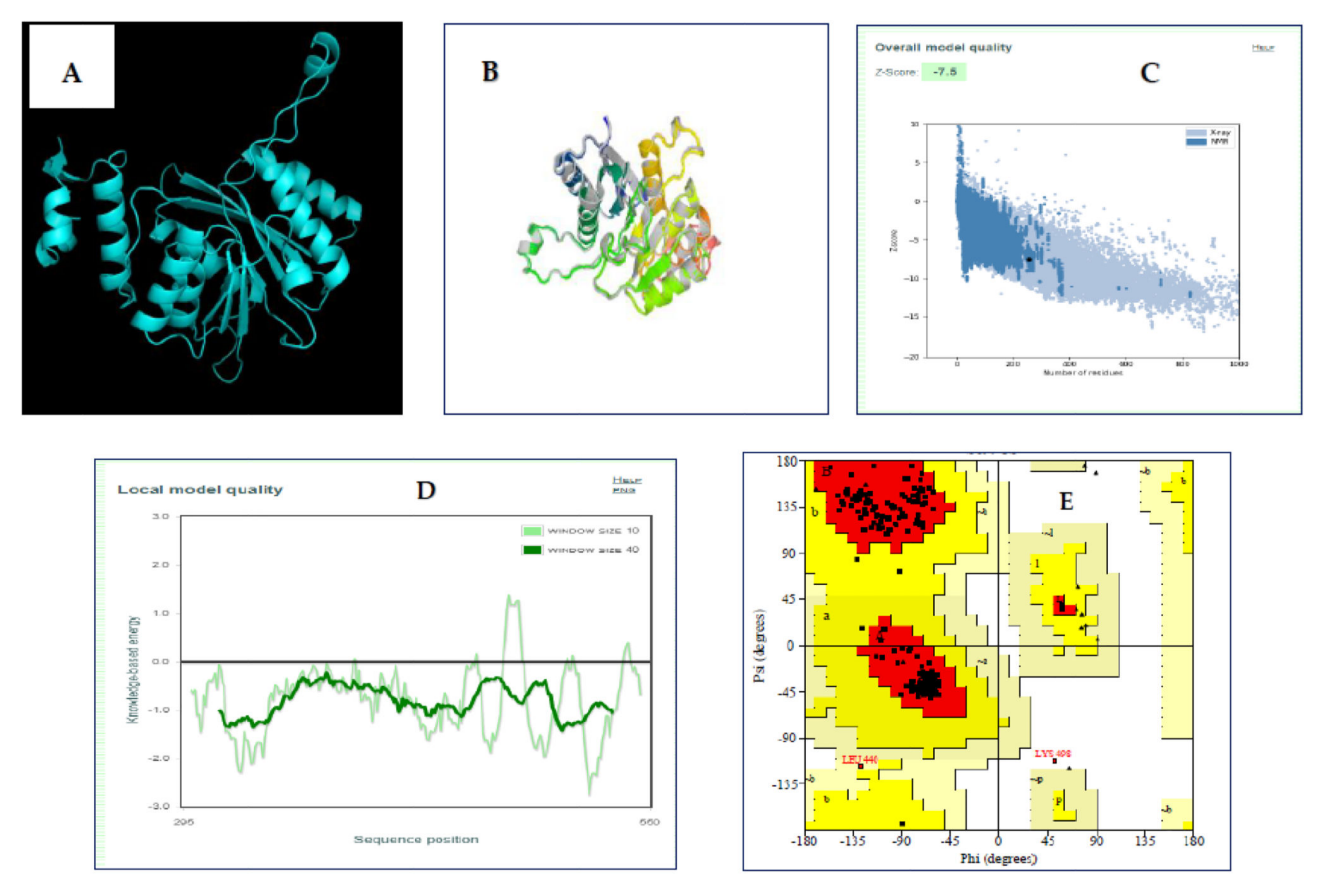
(**A**)Vaccine construct’s 3D crystal structure, (**B**) refined vaccine 3D structure model, (**C**) assessment of the Ramachandran plot for the multi-epitope vaccine construct, and (**D**) a ProSA-web validation of the vaccine 3D structure. The Z-score of the refined model is −7.5, which lies inside the score range (**E**) residue’s score plot by ProSA-web to verify the local model quality.

We utilized phyre2 [44] to complete the buildup of the 3D model structure of the final vaccine construct. The modelled structure based on template c6b92A was predicted by phyre2 to be the best template. This was based on several constructs of the 3D structural model of the vaccine (Figure 6A). In total, 13% (250) of the amino acids residues in the construct were modelled with 100% confidence in a single high scoring template. The GalaxyRefine server [46] was utilized to accomplish the refinement of the 3D structural model of the vaccine construct. The server predicted five (5) refined models. Of these models, model 2 (see Figure 6B) was selected as our final vaccine model because of its quality scores (see Table 5). A yellow highlight depicts predicted B-cell epitopes, which reflects a good surface accessibility. The measurement of similarities between two protein structures is depicted by the global distance test high accuracy (GDT-HA) score [46]. A value of 0.9717 depicts the GDT-HA score. This is a high value, which indicates that there is a high level of similarity between the two models. The root-mean-square deviation (RMSD) score computes the distance between atoms. A low RMSD value depicts a better level of stability. Acceptable RMSD score ranges from 0 to 1.2 [46]. The RMSD score of this model is 0.364. This depicts a good level of protein structure stability. Our vaccine model’s MolProbity score is 1.733 (a value lower than that of the initial model). This shows that critical errors in the 3D model have been reduced. The clash score depicts all unfavorable numbers of overlapping atoms. The model’s clash score was reduced from 48 to 10.6 (an evidence of increased stability to high level). The surface areas of energetically favored regions are depicted by the Ramachandran plot score. An acceptable Ramachandran plot score is that which is greater than 85% [46].

There was an improvement in the Ramachandran plot score from 95.7% to 98.8% after refinement. To validate the overall refined prototype vaccine quality, ProSA-web was utilized. A Z-score of −7.5 was predicted by ProSA (see Figure 6C), which depicts a good quality model.

The local quality of the model is also checked by ProSA. Figure 6D shows the plotted graph of the residue scores. Negative values depict that there is an absence of erroneous parts in the structure of the model. The results of the ProSA-web server presented a Ramachandran plot analysis score of 97.8%, which is like that obtained by GalaxyRefine, which can be found in Figure 6E.

### 3.10 Conformational B-Cell Epitopes Prediction

**Figure 7:**
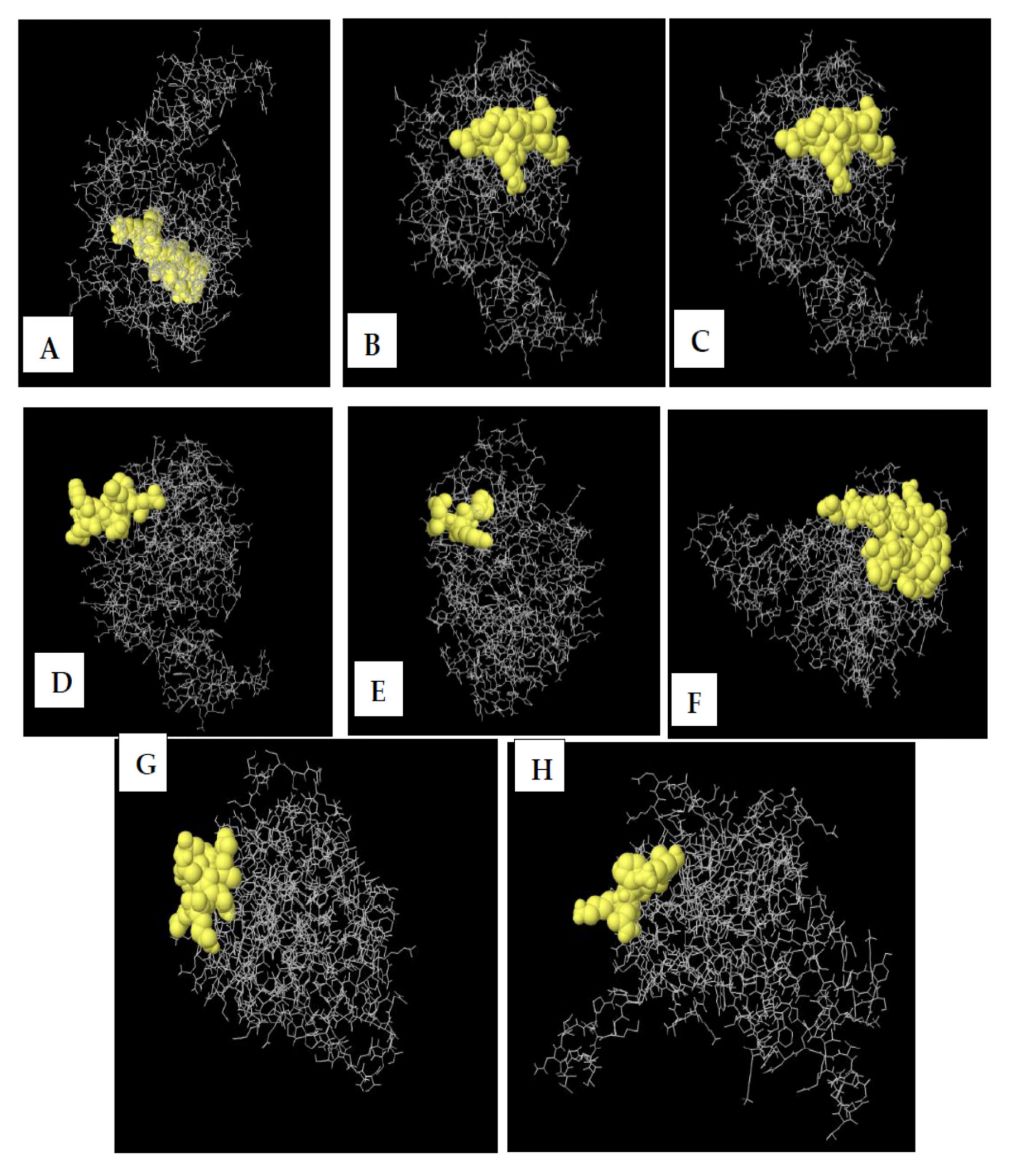
The 3D model of the 8 predicted conformational B-cell epitopes. The yellow regions are the conformational B-cell epitopes, while the grey regions are the residue remnant. (**A**) 18 residues with 0.856 score. (**B**) 32 residues with 0.825 score. (**C**) 12 residues with 0.76 score. (**D**) 11 residues with 0.686 score. (**E**) 4 residues with 0.681 score. (**F**) 19 residues with 0.624 score. (**G**) 8 residues with 0.613 score. (**H**) 6 residues with 0.589 score.

The conformational B-cell epitope of the 3D-refined model was predicted using the Ellipro server [48]. The server predicted eight new conformational B-cell epitopes, which consisted of 110 residues having scores between 0.589 and 0.856. Figure 7 shows the detailed information of the eight epitopes and the 3D model.

### 3.11 Molecular Docking of Vaccine with TLR Receptor

The evaluation of interactions between a ligand molecule and a receptor molecule was conducted through molecular docking. This was carried out to verify the binding affinity of the docked complex. TLR4 was used as the immune receptor for this study to conduct the molecular docking. Toll-like receptor 4 is a very significant human protein that helps with immune response and the recognition of pathogens.

**Figure 8:**
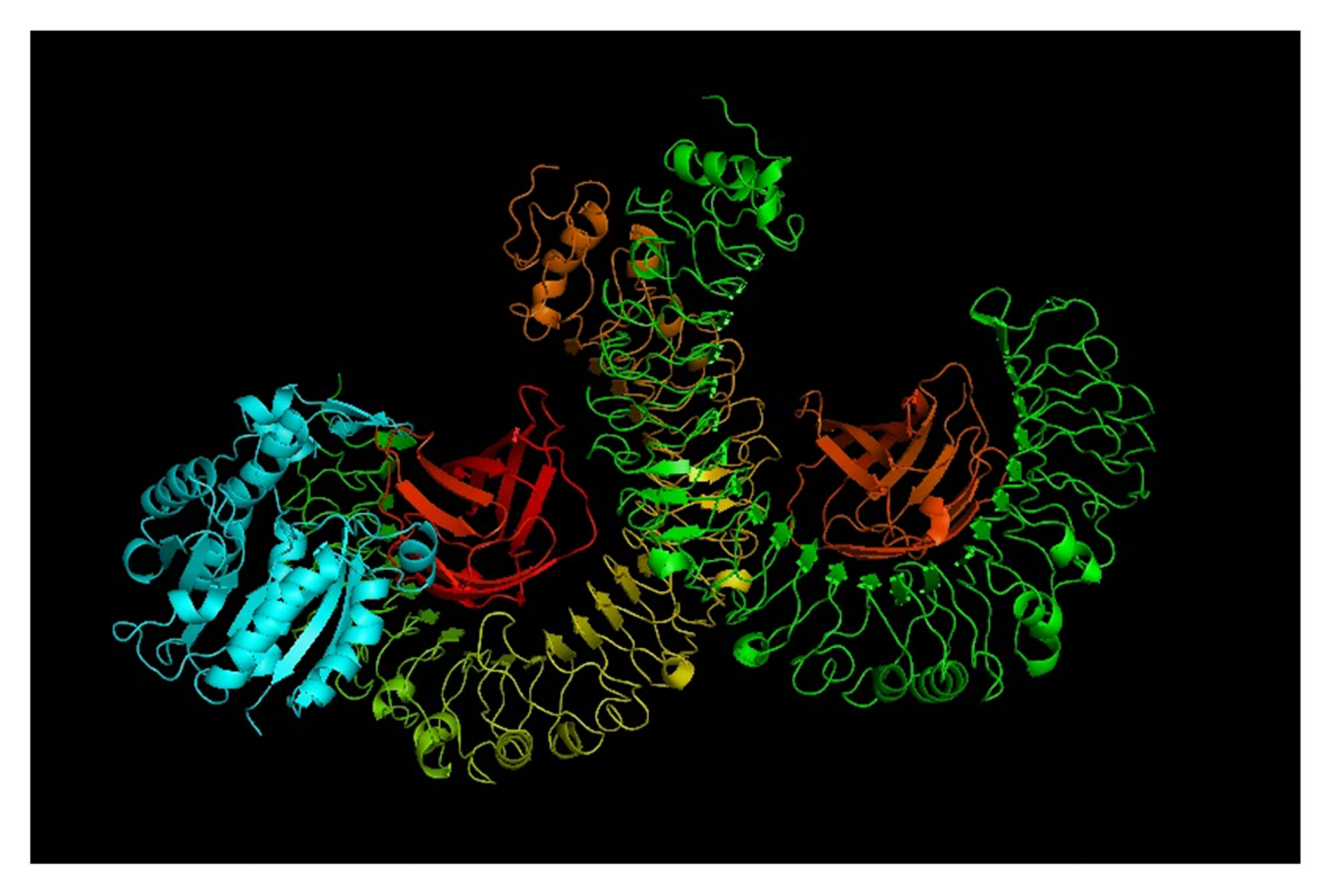
The docked complex of the vaccine model and the TLR4 immune receptor. The vaccine protein is shown in blue, while the rest of the residues are the TLR4 receptor.

Molecular docking was performed by utilizing the ClusPro 2.0 server [49]. Molecular docking was conducted between the final refined 3D vaccine and the TLR4 (PDB ID: 4G8A) immune receptor. Ten different models were produced from the docking process. These models were characterized by low-energy docked structures [49]. A selection was made of the lowest-energy docked model. This result indicates that the vaccine model has good binding affinity and fully occupies the receptor (see Figure 8).

### 3.12 Molecular Dynamics Simulations

**Figure 9:**
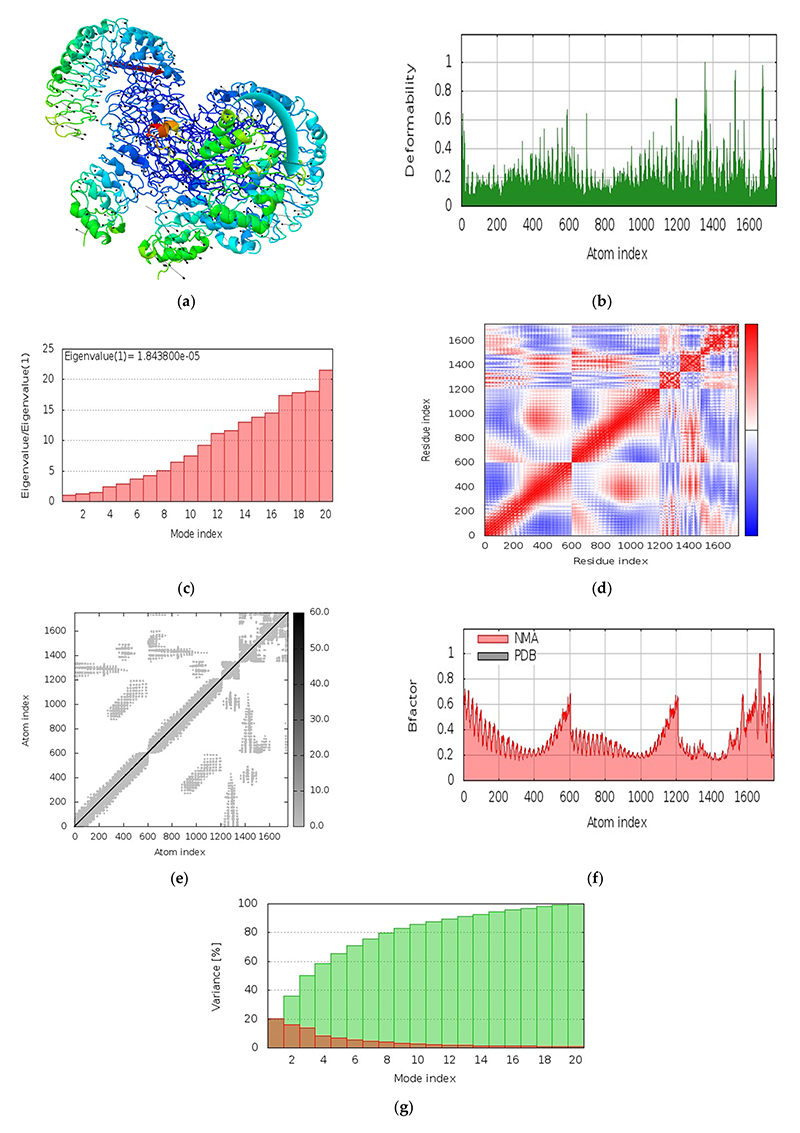
Vaccine–TLR4 complex molecular dynamics simulation, showing (**a**) spin prediction of the ligand–receptor interaction; (**b**) deformability; (**c**) eigenvalue; (**d**) covariance matrix depicting the coupling between pairs of residues (red), uncorrelated (white), or anti-correlated (blue) motions; (**e**) elastic network analysis defining which pairs of atoms are connected by springs; (**f**) B-factor; and (**g**) variance.

The i-Mode server was used to perform analysis of the molecular interaction of the vaccine target with the target TLR-4 receptor. The evaluation of the vaccine–TLR-4 docked complex was carried out by using NMA. The i-Mode suite was used for simulating access to the internal coordinates of the complexity of the system. An examination of the trajectory of the system was performed to determine the deformability. Figure 9 depicts the vaccine–TLR4 complex’s molecular dynamics simulation results showing the spin prediction of the ligand–receptor interaction and other results. The results of the complex trajectory revealed a minimal deformation in the coordinates within the range 0 to 1*°*A. This depicts that the vaccine has a steady binding with minimal deformation (see Figure 9b). Traces of some atomic fluctuations were observed by NMR in the system trajectory of the TLR-4 and the vaccine. Figure 9c shows the computed eigen score of 1.843800 *×* 10^−05^. Furthermore, the covariance matrix analysis revealed the vaccine–TLR-4’s atomic pairs. The analysis depicted the correlated segments in red color, non-correlated segments in blue color, and the uncorrelated segments in white color. Figure 9d shows the integration of the TLR-4 protein residues with the construct of the vaccine and the changes in the TLR-4 binding groove. The model’s elastic network revealed pairs of atomic coordinates through distance-based spring analysis. Each dot in the network plot represents a spring and is colored based on the complex’s stiffness in relation with corresponding atomic pairs. Grey-colored spring models depict the level of compactness and stability of the binding complex system (see Figure 9e). These important results reveal the stable binding and complex rigidity of the vaccine coupled with some atomic fluctuations, characterized with a low deformation index.

### 3.13 Immune Response Simulation

We assessed the vaccine construct’s immune response elicitation by adopting an *in silico* immune simulation technique for 100 steps of simulation. This method is used for the analysis of the capability of the vaccine construct’s immune response elicitation and antigens, amongst others. B cells, T cells, and memory cells that generate immune responses that fight viral infections were assessed by exploring the vaccine candidate. Results obtained from our *in silico* experiments revealed the potency of our designed vaccine candidate. Results revealed that primary and secondary immune responses were elicited through very important players such as the T-cell populations (helper T cells and cytotoxic T cells) and sustainable memory cells (see Figure 11). Figure 11 depicts the induced immune cells as illustrated by the mRNA vaccine.

High measures of IgM, IgG1, and IgG3 antibodies were discovered after administering the vaccine, and prolonged immune responses against the virus were evident through high measures of IgG and IgM immunoglobulins. Furthermore, simulation statistics revealed that an amino acid sequence revealed that cytotoxic T-cells’ elevation, after 13 days of administering vaccine, attained a maximum of 1155 cells per mm^3^. This value gradually decreased to 1120 cells per mm^3^ after 33 days. An increase in helper T cells to 5400–6000 cells per mm^3^ was observed after 5–6 days. An elongated concentration subsisted up to 35 days. Increased immune T cells evoked a high number of memory cells. Adaptive immunity was strengthened against the virus infections because of high levels of HTLs and CTLs in both active and passive states as a response to the vaccine.

Furthermore, there was an increase in the population of the B cells. Similarly, the concentrations of IgM and IgG isotypes increased to around 460–480 cells per mm^3^ and was sustained over a long period. There were also increased levels of cytokines, interleukins, and natural killer cells by the vaccine in *in silico* immunization experimentation (Figure 11). All these results depict the potency and efficacy of the designed vaccine to combat the virus. A mechanism of action for the designed vaccine was proposed. The binding of mRNA vaccine to MHCs and TLR receptors activates key players against the virus (Figure 11). After vaccine administration, there was proliferation of HTLs, CTLs, and other regulatory immune cells to destroy the virus.

## 4 Discussion

The outbreak of the SARS-CoV-2 virus is a major global pandemic [55]. Prior the evolution of vaccines in past decades, vaccines have brought about the complete extermination or near eradication of some infectious diseases [34]. These diseases include measles, rubella, smallpox, mumps, polio diphtheria, pertussis, and tetanus [56]. Vaccination has been the most successful and effective public health strategy adopted for the eradication of various infectious diseases. Presently, vaccination has become an effective tool for preventing diseases and drastically reducing the negative impacts of different dreaded diseases.

However, the increased transmission of COVID-19 (SARS-CoV-2) has resulted in millions of deaths worldwide and caused wreckage on the economies of many nations of the world [57]. These therefore call for the development of effective and safe prophylactics or therapeutics that could be administered to either mitigate the effects of the menace caused by the deadly virus or protect against its ever evolving and mutating new variants. Several methods have been devised to develop an effective medical therapy, such as vaccines to prevent virus transmission; however, many of the methods have been quite laborious and time-consuming and may ultimately slow down efforts in the development of an effective vaccine, thus contributing less towards mollifying the recent spread of the disease [57,58].

One of the most popular approaches adopted in the past is the conventional method of vaccine design such as live attenuated and inactivated viral vaccines, which utilizes the traditional vaccine development pathway, based on cultivation and inactivation of pathogenic organisms. Although this approach has successfully provided an enduring protection against infectious diseases, mRNA vaccines nonetheless possess great promise for the future [59], as they have been proven to have many merits over conventional vaccine platforms [60]. Besides safety and potency, one of the important benefits of mRNA is the flexibility of its design. Its antigen-coding sequence (open reading frame, ORF) can be easily modified at specific locations and/or codon-optimized to bring about improvements in translation or help channel antigens to the right compartment [60]. Our study was centered on designing an mRNA vaccine against COVID-19 using an array of bioinformatics and immunoinformatics tools to predict epitopes inducing the immune system.

The first step in the design of a novel prophylactic and immunotherapeutic vaccine involved predicting the T-cell and B-cell epitopes. Identifying epitopes is a very important process for the development of effective antibodies that can help neutralize bioactive proteins. Identifying the correct epitopes helps to select high affinity antibodies for immunotherapy and immunodiagnostics [61]. T-cell epitopes are very important for the purpose of adaptive immune simulation, and they interact with MHC molecules [33]. Therefore, when selecting an mRNA vaccine, it is expedient to ensure that a target is immunogenic and can elicit a protective immune response [62].

In this study, highly immunogenic epitopes for B and T cells, humoral prime molecules, and immunity as mRNA vaccine candidates were determined to combat COVID-19 disease. Checking through the various parameters, five CTL epitopes, one HTL epitope, and one LBL epitope were extracted and connected by using the EAAKEAAK and AAY linkers (see Table 1). EAAKEAAK and AAY linkers were connected between the selected epitopes to enable a rational design of mRNA vaccine. The GPGPG linker was also embedded between the adjuvant and the epitope sequences to produce bioactivity improvement for the vaccine [33].

During vaccination, quite a reasonable amount of antibody and T-cell responses are produced, and these required administration of multiple doses of the purified antigens to elicit sufficient antibody response [63]. To address these challenges, significant efforts have been put in by researchers to identify components defined as adjuvants capable of increasing the immunogenic response of antigens in vaccines. Adjuvants are very important in increasing the potency and efficacy of a vaccine [64]. Incorporating them into vaccine design has many advantages, which include provision of stronger immune responses [63]. In this study, we included the co-stimulatory molecule CD40L as our adjuvant. Its involvement in this study was considered due to its inherent efficiency to stimulate the professional antigen-presenting cells (pAPCs), which could invariably lead to the induction of immune response molecules. Although several studies have revealed that mRNA possesses a self-adjuvanting property when administered naked, including an adjuvant will nevertheless contribute more to its efficacity. CD40L is a cell-surface interaction molecule whose expression is pronounced in a CD4+ T-cell subset [65].

Shortly after activating the T cells, its expression is induced. This depicts an early activation marker of T lymphocytes. After a careful and detailed study of its pathway, it was revealed that CD40L plays multiple roles in ensuring a healthy immune system. These include enhancing antigen-specific T-cell response by activating the dendritic cells and the induction of interleukin 12 (IL-12) production by the cells [65].

This response could be sustained for the duration of time the antigen’s presence is felt within the system and the time it takes to interact with CD40+ target cells [65].

The previous mRNA approaches in the design of vaccines have produced remarkable results in the past decades. Although there have been shortcomings in their production, notably in their stability and delivery [54,66] in the production of an RNA vaccine, stability and translation of mRNA is crucial [67]. The fact that the half-lives of mRNA molecules are relatively short and tend to be easily degraded in the body calls the need for improvement on the mode of mRNA vaccine production before its administration for proper stability and the efficient promotion of mRNA therapy [68]. 5′ and 3′ UTRs Five prime and three prime UTRs were incorporated into the vaccine ORF to ensure the sufficient production of antigens and effective vaccination of host. The 5′ untranslated region, or 5′ caps, carry out efficient protein production, while the 3′ untranslated region determines mRNA stability and increased protein translation [67].

The frequent transmission of SARS-CoV-2 across the globe has created a platform for making its RNA sequence subsequently undergo mutations, which invariably lead to the translation of different viral proteins (Zikun et al., 2021). Although, these types of mutations can have influence on the epitope-based vaccines, because a change in a single amino acid can alter the results predicted from the epitope analyses (Zikun et al., 2021). However, the proposed final vaccine candidate can tackle the mutations. A multiple sequence alignment was performed for 100 randomly selected SARS-CoV-2 spike glycoproteins from the study area. The results obtained showed no occurrence of mutation in the selected epitope area (Figure 2), thereby indicating the effectiveness of our vaccine construct.

In the context of genetically heterogeneous human populations, HLA polymorphism and its consequent population coverage has been the major concern in epitope-based vaccine design. To address the situation, a careful consideration of the population coverage of the T-lymphocytes epitopes is needed because individuals will likely react to different sets of peptides from a given pathogen [69]. The coverage of the CTL and HTL epitopes was assessed to predict the vaccine construct’s effectiveness within the study areas. The epitopes showed a good population coverage (77.22% in average), and a high degree of coverage was predicted for all the regions under study (Table 3), thus indicating the possibility that the vaccine construct can promote an immunological reaction within the population in the study areas. These high values are needed to reduce the complexity of including different epitopes in the vaccine development.

The physicochemical properties of the vaccine construct prove its ability to be a good potential candidate for a vaccine. The molecular weight (MW) was 223.1 kDa, higher above the acceptable threshold value of 110 kDa for a good vaccine candidate (Chukwudozie et al., 2021), thus signifying the efficacy of the vaccine construct. The estimated theoretical pI was 8.69, suggesting the vaccine to be slightly basic. The score of the instability index (48.78) is slightly above the standard threshold. This suggests that the protein would be unstable upon expression, therefore validating the problem of instability, which is majorly encountered in the production of mRNA vaccines. The aliphatic index shows that the vaccine construct is thermostable. The GRAVY score obtained (−0.296) proposes that the vaccine construct is hydrophilic, representing its ability to be highly soluble upon expression as seen in Table 4.

In the development of a vaccine, having the knowledge of the secondary and tertiary structure of the target proteins is crucial to gaining a better understanding of the constructed vaccine candidate. The analyses of our vaccine’s secondary structure revealed that the protein contained mainly 33.38% coils. Secondary structures have been shown to be recognized by a few innate immune receptors, and this recognition most times tends to inhibit protein translation. To avoid being recognized by these immune receptors, incorporation of modified nucleosides, such as 5-methylcytidine (5 mC) and pseudouridine (ψ), optimized codons, and a cap-1 structure are important, as it may in turn improve the efficiency of the protein. The 3D structure improved well after refinement. The Ramachandran plot indicates that 97.8% of the residues lie within the favored regions, and 1.3% are allowed regions with less (0.4%) residues in the outlier region. This has provided more evidence that the model’s quality is acceptable.

The analysis of antibody–antigen interactions is a very important modeling and docking concept required in vaccine design [49].

The application of protein docking is essential to determine the structure of the antibody–antigen complexes. This interaction is very crucial in understanding the basic function of cells and larger biological systems in all living organisms [70]. To determine the ability of the vaccine construct to bind with TLR on immune cells, TLR-4 was docked with the vaccine considering its importance for easy recognition of pathogens and stimulation of immune response. The results revealed a constructed vaccine with high binding affinity towards the TLR-4. This interface of vaccine with TLR-4 significantly indicates the probability of the vaccine to have the potential of stimulating innate and adaptive immune response. Subsequently, in an advent to explore the stability and dynamics performance of the TLR-4–vaccine docked complex; a molecular dynamic simulation was conducted. The RMSD plot depicts a steady binding of the complex.

The *in silico* immune response simulation depicted a consistency in the immune response. There was an increase in the generated immune responses before its repeated exposure to the antigen. There was evident development of memory B cells and T cells, with memory in B cells lasting several months. This indicates humoral immunity and is essential for complimenting the immune response. Moreover, helper T cells were particularly stimulated, therefore establishing the capacity of the vaccine construct to protect against SARS-CoV-2.

The negative impacts of COVID-19 cannot be overemphasized. Such impacts have affected the economy of many countries [71]; stock markets have been affected by COVID-19 [72,73]; COVID-19 outbreaks have affected the mental health of many categories of people [74]; it has had a negative impact on public health and on addressing non-COVID-19 diseases [75,76]; the COVID-19 pandemic has negatively impacted on health workers by causing anxiety and high levels of stress [77,78]; COVID-19 has had mental and psychological impacts on different individuals [79–81].

Prior the emergence of the SARs-CoV-2 virus, several bioinformatics, computational informatics, and modeling approaches have been applied towards proffering solutions to existing infectious and non-infectious diseases such as HIV, Ebola, malaria, and hereditary diseases, amongst others [82–91]. In this current work, bioinformatics, computational, and modeling approaches are being harnessed towards developing a potent and effective vaccine candidate against SARS-CoV-2 virus.

Data collection within the COVID-19 pandemic has been from different (diverse) sources. Real-time dashboard COVID-19 data have indicated or revealed the impact of COVID-19 on human health and human lives [92–95]. Some of the sources are in real time, for instance web-dashboards [92–95], while others are diverse. COVID-19 data are of different forms, namely genomic sequences of different variants of the SARS-CoV-2 virus [92–95], chest X-rays of COVID-19 patients [96], kidney replacement therapy data for COVID-19 patients [97], lungs data of patients [98], blood data of COVID-19 patients [99–104], medical data of patients that were infected and recovered [105], medical data of patients that were infected and died [106], patients’ demographic data, patients’ bio-data, COVID-19 health facility data for different regions of the world, and COVID-19 phylogenetic data, amongst others. Genomic data collected for the SARS-CoV-2 virus were stored in bioinformatics databases such as NCBI, EBL, and GISAID [20,107,108].

To examine the binding stability, conformation, and interaction modes of the vaccine–TLR4 docked complex, the molecular dynamics were simulated using the online WEBGRO macromolecular simulations platform [109]. WebGro is an entirely automated online tool for simulating macromolecules (proteins) alone or in complexes with ligands (small molecules) using molecular dynamics modeling [109]. For comprehensive solvated molecular dynamics simulations, WebGro utilizes the GROMACS simulation program [110]. The energy of the complex formed was first minimized using the steepest descent integrator at every 5000 steps for molecular dynamics simulation. Afterwards, enough of an amount of Na+ and Cl- counter ions were added to maintain a salt concentration of 0.15 M in the complex system. The NVT/ NPT equilibration was performed at 300 K and 1 bar pressure. In addition, leapfrog was selected as the MD integrator for a simulation time of 100 ns and 1000 frames per MD simulation [110]. To better understand the formation of the complex, a trajectory analysis of the root-mean-square deviation (RMSD), root-mean-square fluctuation (RMSF), radius of gyration (Rg), solvent accessible surface area (SASA), and hydrogen bonds (HBs) was performed [110].

**Figure 12:**
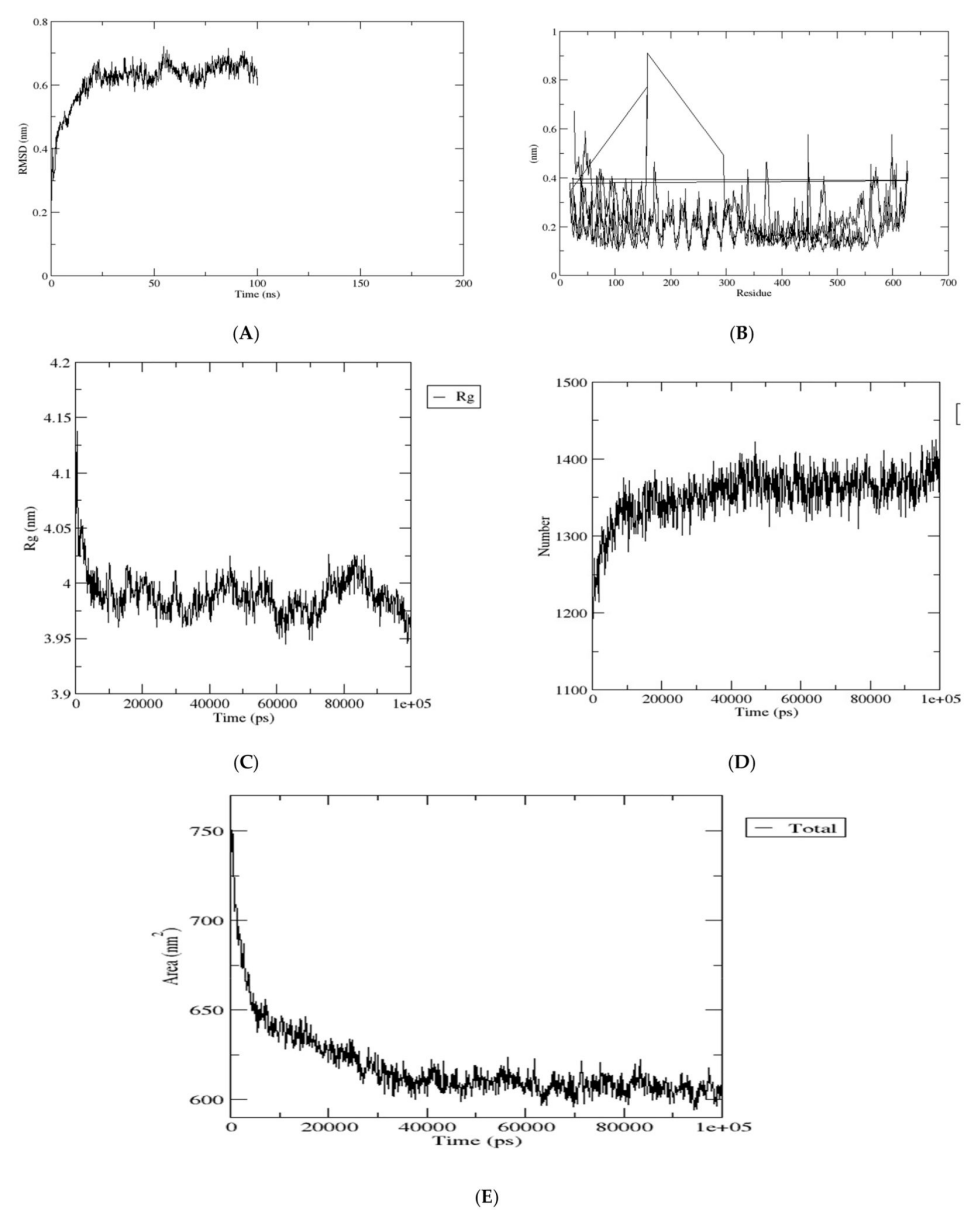
(**A**) RMSD study plot for 100 ns MD simulation of vaccine–TLR. (**B**) RMSF study plots for 100 ns MD simulation. (**C**) Radius of gyration study plot for 100 ns MD simulation vaccine–TLR. Solvent accessible surface area study plot for 100 ns MD simulation of vaccine–TLR. (**E**) Solvent accessible surface area study plot for 100 ns MD simulation of vaccine–TLR.

One of the main functions of the root-mean-square deviation (RMSD) is to depict the average distance between the backbone atoms of the starting structure and the simulated structures when superimposed [111]. As a useful parameter, the RMSD can be utilized to study the equilibration of MD trajectories as well as checking the stability of complex systems during simulation. This could be achieved by plotting the RMSD of the protein backbone atoms against time to see how the structural shape of the protein changes over time [111]. The RMSD plot was significantly dynamic, with fluctuations occurring after 5 ns. The stable conformation was attained at a time range between 75 and 100 nanoseconds, with no significant changes in the results (see Figure 12A).

Furthermore, the RMSF values of the protein atoms were calculated and plotted against the residues. When evaluating the stability and flexibility of a complex system, another important parameter to consider is the root-mean-square fluctuation (RMSF).

This parameter is useful because it can be used to study how well the behavior of amino acid residues in a target protein changes as it binds to a ligand [111]. Throughout the simulation, the amino acid showed very little fluctuation (Figure 12B). Additionally, the complex systems’ gyration radius (Rg) was investigated. This is the distance between the rotational axis and the mass center [112]. It is essential and important to know and understand how structural variation affects the compactness of the protein after binding with the ligands when examining the stability and flexibility of the complex structure during simulation [113], and this can be accomplished by analyzing the complex structure’s radius of gyration (Rg). Higher Rg values indicate that the protein is less compact and flexible, whereas low values indicate that the protein packing has not changed much (see Figure 12C), thus exemplifying the high degree of compactness and stiffness. To investigate changes in structural compactness, the Rg values of protein backbone atoms were plotted versus time. The backbone Rg values gradually declined until they reached 10 ns. There were no significant variations in the time between 11 and 100 ns, and a nearly constant value of about 4.0 nm was maintained, indicating that the protein packing did not vary considerably.

Similarly, we examined the formation of hydrogen bonds in the complex structure by plotting the number of hydrogen atoms against time. This is necessary for a better understanding of the protein’s structural integrity, catalytic region, and protein–ligand interaction in the complex structure [113]. Within the complex structure of the protein, there is a significant change in the hydrogen bond interaction (see Figure 12D).

We also calculated the interaction area between the solvent and the protein complexes to implement the solvent accessible surface area (SASA) of the complex structure. To assess changes in surface area, the protein’s values were plotted against a function of time. SASA is a significant parameter for determining the extent of receptor exposure to surrounding solvent molecules during simulation [111]. SASA with a higher value indicates more hydrophilicity [113]. The SASA complex trajectory values gradually decreased till 400 ns. Throughout the simulation period, minute changes were noticed, except for a few time intervals (see Figure 12E). The average SASA value was 610 nm^2^, with values ranging from 625–600 nm^2^.

## 5 Conclusions

In conclusion, the process involved in the production of an effective traditional vaccine often takes several months or years of trial before it can be accomplished. Moreover, these vaccines are quite expensive. The integration of the bioinformatics approach into the development of vaccines has helped overcome many of these challenges by focusing mainly on the selection of appropriate antigens or antigenic structures, carriers, and adjuvants used in the design. In the face of the current pandemic, which has ravaged the world, the development of vaccines is an urgent need. This is especially true for African countries, which lack critical infrastructure for vaccine development to combat the circulating variants within the region. Our results show that the vaccine candidate consisted of seven epitopes, namely a highly immunogenic adjuvant, an MHC I-targeting domain (MITD), a signal peptide, and linkers. The vaccine candidates’ molecular weight (MW) was predicted to be 223.1 kDa, which is greater than the acceptable threshold of 110 kDa on an excellent vaccine candidate. The summary of the results obtained from the experiments revealed that the vaccine candidate was antigenic, non-allergenic, non-toxic, thermostable, and hydrophilic. The vaccine candidate has good population coverage, with the highest range in East Africa (80.44%) followed by South Africa (77.23%). West Africa and North Africa have 76.65% and 76.13%, respectively, while Central Africa (75.64%) has minimal coverage. Evaluation of the secondary structure of the vaccine construct revealed a stabilized structure showing 36.44% alpha-helices, 20.45% drawn filaments, and 33.38% random helices. Molecular docking of the TLR4 vaccine showed that the simulated vaccine has a high binding affinity for TLR-4, reflecting its ability to stimulate the innate and adaptive immune response. Bioinformatics, computational, and immunoinformatic approaches for a multi-epitope mRNA vaccine design against the circulating variants of SARS-CoV-2 within the African population have shown that this vaccine candidate can be a useful therapeutic in fighting the deadly virus. This is because the designed construct has been shown to meet the requisite threshold for each of the physicochemical properties that make a candidate vaccine effective. According to our findings, the designed construct is antigenic, non-toxic, non-allergenic, slightly basic, thermostable with wide population coverage, and capable of tackling any mutation. Further work can be carried out after the results and performances from this computational research have been subjected to in vitro and in vivo validations.

## Figures and Tables

**a – f:**
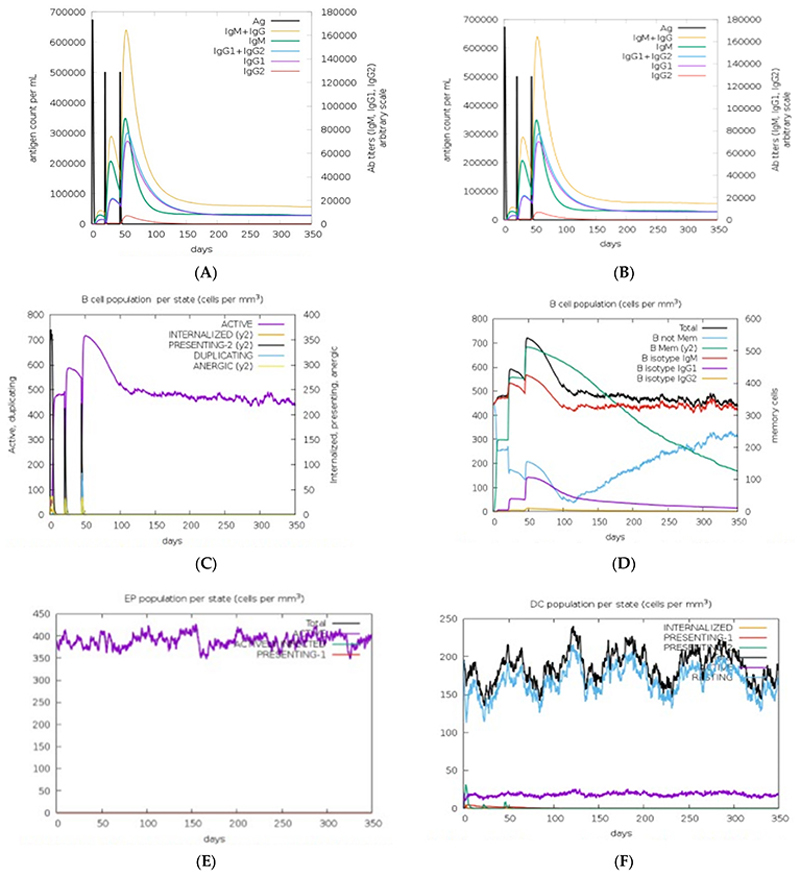


**g – l:**
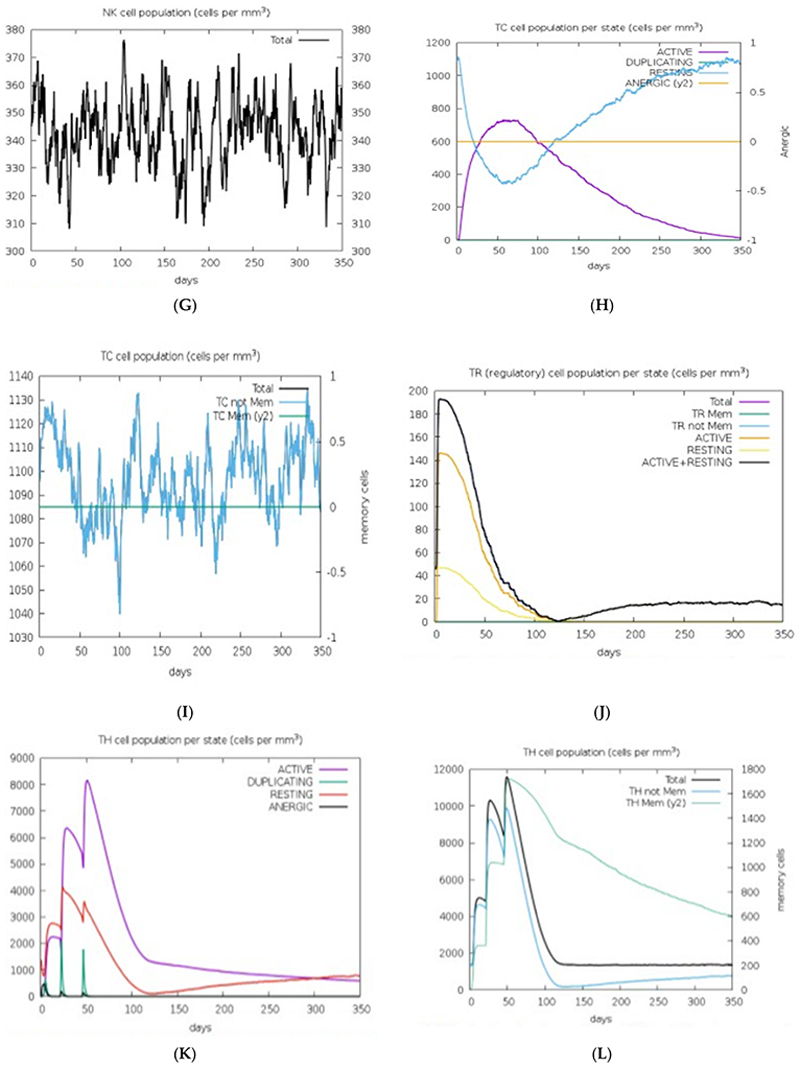


**m – o:**
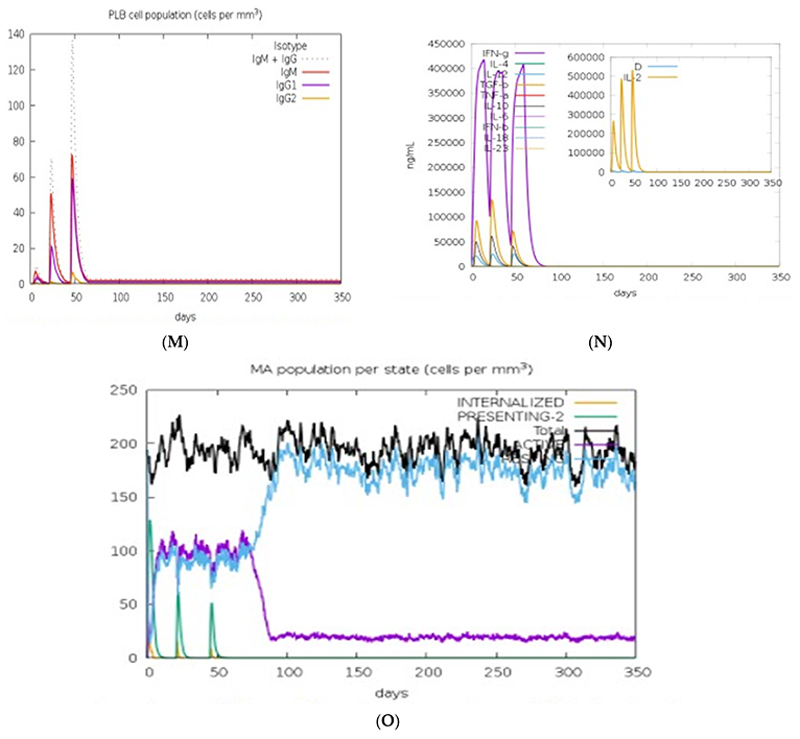


**Table 1:** Epitopes selected for vaccine construction.

Recognizing Cell	Epitope Sequence
	WTAGAAAYY
	HRHLRFLTL
Cytotoxic T lymphocyte	YQPYRVVVL
	YPQILLLVL
	SPRRARSVA
Helper T lymphocyte	ISFHVLTKLRLKCKL
B lymphocyte	WVFITTKTTKVGWKVSSEF

**Table 2:** Selected T-lymphocyte epitopes and their associated MHC alleles.

T-Lymphocyte Type	CTL Epitopes	MHC Binding ALLELES
CTL	WTAGAAAYY	HLA-A*29:02, HLA-A*30:02, HLA-B*15:01, HLA-B*46:01, HLA-B*58:01, HLA-B*53:01, HLA-B*35:01, HLA-C*07:01, HLA-C*03:03
	HRHLRFLTL	HLA-B*48:01, HLA-C*06:02, HLA-C*07:01
	YQPYRVVVL	HLA-A*02:06, HLA-A*32:01, HLA-B*48:01, HLA-B*46:01, HLA-C*06:02, HLA-C*07:01, HLA-C*03:03
	YPQILLLVL	HLA-B*51:01, HLA-B*53:01, HLA-B*35:01
	SPRRARSVA	HLA-B*51:01
HTL	ISFHVLTKLRLKCKL	HLA-DRB1*11:01

**Table 3:** IEDB server predicted results.

Population/Region	MHC Class Combined		
	Coverage Area	Average Hit	PC90
Central Africa	75.64%	2.21	0.41
East Africa	80.44%	2.33	0.51
North Africa	76.13%	2.29	0.42
South Africa	77.23%	2.23	0.44
West Africa	76.65%	2.22	0.43
Average	77.22	2.26	0.44
Standard deviation	1.7	0.05	0.04

**Table 4:** Allergenic, antigenic, physicochemical assessments, and toxicity of the vaccine construct.

Features	Result	Assessment
Number of amino acids	1995	Suitable
Molecular weight	223.1 kDa	Average
Theoretical pI	8.69	Slightly basic
Total number of negatively charged residues (Asp + Glu)	196	-
Total number of positively charged residues (Arg + Lys)	223	-
Total number of atoms	312178	-
Chemical formula	C_9908_ H_15532_ N_2774_O_2906_S_97_	-
Instability index (II)	48.78	Unstable
Aliphatic index	82.13	Thermostable
Grand average of hydropathicity (GRAVY)	–0.296	Hydrophilic
Antigenicity	0.5059 (VaxiJen) 0.7334 (ANTIGENPro)	Antigenic Antigenic
Allergenicity	Probable non-allergen (AllerTOP 2.0 and AllergenFP)	Non-allergen
Toxicity	Non-toxin (ToxinPred)	Non-toxic

**Table 5:** Predictions of the models’ quality scores by GalaxyRefine.

Model	GDT-HA	RMSD	MolProbity	Clash Score	Poor Rotamers	Rama Favored
Initial	1.0000	0.000	2.457	48.0	0.0	95.7
Model 1	0.9707	0.353	1.527	10.2	0.4	98.4
Model 2	0.9717	0.364	1.733	10.6	1.8	98.8
Model 3	0.9658	0.371	1.570	11.3	0.4	98.8
Model 4	0.9707	0.365	1.594	12.1	0.0	98.4
Model 5	0.9697	0.359	1.545	10.6	0.4	98.4

## Data Availability

Experimental data sequences for this research are freely available but can be found in the GISAID database (https://www.gisaid.org/); Access date: 30 March 2021. GISAID data can be shared only with GISAID registered database users and not with the non-registered. All relevant data for this article are within the manuscript and its Supporting Information files. This project information can be found at: https://github.com/oluwagbemi/Bioinformatics-and-Computational-Approaches-to-the-Design-of-mRNA-COVID-19-vaccine-candidates-. This project’s information can also be found on the research project page with weblink: https://olugbengaoluwagbemi.weebly.com/research-projects.html.
